# Optical strain based pre-failure indication in failure process of geomaterials

**DOI:** 10.1038/srep24741

**Published:** 2016-04-21

**Authors:** Sudipta Bhattacharjee, Debasis Deb

**Affiliations:** 1 Advanced Technology Development Center, IIT Kharagpur, 721302 India; 2 Department of Mining Engineering, IIT Kharagpur, 721302 India

## Abstract

Multilevel extended digital image correlation (X-DIC) technique based on finite element method (FEM) is applied for measuring deformation of geomaterials under uni-axial loading condition. The concept of Smooth Particle Hydrodynamics (SPH) is introduced for smoothing computed displacements as well as for calculating strain tensors at every nodal point of FEM mesh. Cumulative effective strain estimated from strain tensors is found to be a well suited parameter to identify the change in stress-strain behaviour in experimented samples. Further analysis suggests that onset of microcrack development and yielding in samples can also be identified using this parameter. Based on these findings, an indicator is developed for determining onset of both microcrack development and yielding in geomaterials. This indicator is found to be related to volumetric strains and may also signify dilation of the sample. The potential of the developed indicator is tested by conducting four experimental works with concrete and rock samples.

Generally rock failure process contains several distinct deformation stages^1^. Stress-strain behaviour of geomaterials is of utmost important for stability of structures made for civil, mining and other engineering applications. One of the aspects of this behaviour is to identify the yield point, where material departs from elasticity. Over the years, many contact based sensor technologies have been tried for determining threshold, and/or indicator of failure in rock and concrete under different loading conditions.

Lei *et al.*^2^ have investigated the spontaneous fault nucleation and its quasi-static growth in intact brittle rocks under triaxial compression by monitoring acoustic events (AE) during failure process^2^. In Gale *et al.*^3^ have tried to correlate rock failure with microseismic events to reduce ground control hazards in underground mining^3^. Frid and Vozoff^4^, have conducted high frequency electromagnetic radiation (EMR) study to generate early warning signal during rock failure process^4^. It has been found that, indicators of critical point prior to rock failure can be inferred from pre-failure damage^5^. In 2010, the failure process of violent rock has been studied by He *et al.*^6^. It has been found that, the dynamic unloading test provides an useful information to gain an insight into the characteristics of rock bursts. Niccolini *et al.*^7^ have applied accelerometric transducer for detecting acoustic elastic emission (ELE) signal during damage monitoring in rocks and concrete^7^.

The methods discussed above provide reasonable insight into the failure process however measurement may be disrupted if contact between the sample and sensor becomes loose. In addition, it is also not possible to monitor status of the sample with time at every location in a structure and hence vital information regarding the failure process may not be recorded for further investigation. On the other hand, the sensitivity and accuracy of sensors inside a sample may also degrade with time.

Digital image correlation (DIC) is an established non-contact technique for monitoring deformation/strain of an object under loading conditions. Many pioneering works have been carried out for monitoring of deformation or strain of object/structure. In Sun *et al.*^8^ have presented a finite element formulation for a digital image correlation method that directly determine two-dimensional displacement fields during the image correlation process with digital images^8^. It has been observed that the image correlation with the finite element formulation is computationally efficient, accurate, and robust. Quinta da Fonseca *et al.*^9^ have presented the fundamental aspects of optical correlation with emphasis on the applicability of the technique to the analysis of micrographs obtained during *in situ* deformation studies^9^. They have analysed image sequences obtained during *in-situ* deformation tensile tests on two very different materials: antler bone and ferritic steel. In Yao *et al.*^10^ have applied the digital speckle correlation method (DSCM) for the full-field deformation measurement of carbon fiber/epoxy composite pressure vessel^10^. They have evaluated the full-field displacement and strain distribution of a composite pressure vessel under internal pressure and the results are verified by using conventional electronic strain gauge technology. Besnard *et al.*^11^ have proposed a methodology to estimate displacement fields from pairs of images (reference and strained) that evaluates continuous displacement fields^11^. In Yoneyama *et al.*^12^ have applied the concept of DIC for deflection measurement of the real bridge^12^. The concept of extended finite element (X-FEM) method has been applied by Rethore *et al.*^13^ for the measurement of displacements using digital image correlation^13^. DIC technique has applied by Huang *et al.*^14^ for real-time monitoring of clamping force during screw fastening^14^. The concept of X-ray micro tomography imaging with three-dimensional volumetric digital image correlation techniques have been combined by Hall *et al.*^15^ to observe and quantify the onset and evolution of localised deformation processes in sand with grain-scale resolution^15^. In Koerber *et al.*^16^ have investigated the effect of strain rate (up to 350 *s*^−1^) on polymer-based composite materials by conducting quasistatic and dynamic experiments^16^. DIC method has further refined by Poissant and Barthelat^17^ by developing a novel subset splitting procedure on discontinuous displacement fields^17^. In Chen *et al.*^18^ have proposed two-step X-DIC to measure full-field displacement with discontinuities by using the partition of unity method based on X-FEM^18^. Nguyen *et al.*^19^ have analysed the fracture evolution from inclined flaws (cuts) in a soft rock deformed under plane-strain uniaxial compression using X-DIC method which provides experimental quantification of fracture mode (opening/closing and shearing)^19^. In Rechenmacher *et al.*^20^ have employed DIC technique to identify and characterize the development of vortex structures inside shear bands formed in dense sands during plane strain compression^20^. Son *et al.*^21^ have performed the three-dimensional digital image correlation (3D DIC) analysis to investigate the displacements on the surface of a dense sand specimen during a triaxial compression test^21^. To achieve the objective, they have developed a 2D finite element model for comparing the experimental results under displacement controlled loading conditions and found to be in good agreement. Bhattacharjee and Deb^22^ have demonstrated that multilevel FEM-DIC method provides one order higher accuracy as compared to subset based DIC technique.

All of the above literatures demonstrate that DIC technique is well equipped to estimate displacement fields of an object in a non-contact manner. Deb and Das^23^ have successfully implemented X-FEM for analysis of displacements and strains in a jointed rock sample. Based on this approach, multilevel X-DIC method is implemented to capture high precision displacement in deformed images due to existence of a discrete discontinuity^24^. The accuracy of estimating strain at nodes can be improved by smoothing the computed displacement then applying a particle based approach for calculating spatial derivatives. The concept of Smooth Particle Hydrodynamics (SPH) is applied in this study for estimating strain tensors at nodes. This paper describes the application of proposed method using four experimental works. For this purpose, a system is designed to acquire image, load and platen displacement data synchronously under uniaxial loading condition.

Based on the strains obtained between two consecutive images, cumulative effective strain (*ε*_*eff*_) is determined at nodes with passage of time or increment of loading or axial strain. The trend of *ε*_*eff*_ with axial strain reveals that it is possible to derive a precursor to determine onset of yielding during failure process of geomaterials^25^. The results are also compared with the concept of yield point proposed by Mogi^26^. In this paper, an algorithm is developed for monitoring of sample failure mechanism in real time manner and for generating yield point during material failure process. The paper also enumerates the physical significance of developed indicator which signifies volumetric strain that may develop in samples. Four experimental works are presented in the paper to show the efficacy of the developed prefailure indicator for identifying yielding and microcrack development in samples in a non-contact manner.

## Methods

### X-DIC method

X-DIC is a method which primarily establishes a displacement field of pixels of a deformed image with a discontinuity with respect to a reference or undeformed image of the same object with or without the discontinuity. Let us consider two gray scale images, having intensity function *f*(**x**, *t*) for a reference image at time *t* and *g*(**x**, *t* + Δ*t*) for the deformed image at time *t* + Δ*t* such that conservation of optical flow occurs as


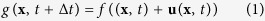


where **u**(**x**, *t*) denotes displacement field in between *t* and *t* + Δ*t*. Assuming *f*(**x**, *t*) is only spatially differentiable (considering two consecutive images) and then, applying the first order Taylor series expansion of *g*(**x**) with respect to *f*(**x**), yields^13^


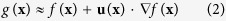


Therefore, the quadratic residual *ψ* of the entire domain can be written as


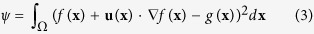


Now, we assume that the domain Ω is cut by a crack or joint. Hence equation 3 can be rewritten as^24^:


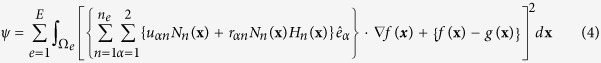


where *n*_*e*_ is the number of nodes per element, *u*_*αn*_ and *r*_*αn*_ are the unknown regular and enriched nodal displacements respectively at node *n* in dimension *α*, *e*_*α*_ is the unit vector of dimension *α* and *N*_*n*_(**x**) is the nodal point shape function. The function *H*_*n*_(**x**) is the Heaviside step function at node *n*.

Equation 4 is minimized with respect to nodal point displacements *u* and *r* separately and the following linear system of equations is obtained for each element^24^.


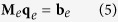


where


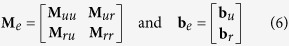


The elements of the matrices **M**_*e*_ and **b**_*e*_ are given by


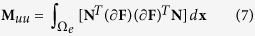



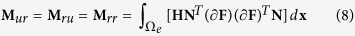



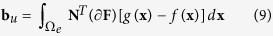



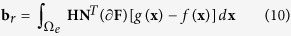


In practice, derivatives are obtained by averaging contribution from both reference and deformed images as ∂**F** ≈ 0.5[∂**F** + ∂**G**]. In this paper, both *f*(**x**) and *g*(**x**) are interpolated with cubic B-spline functions in two dimension^27^.

After estimating elemental **M**_*e*_ and **b**_*e*_, they are assembled globally using standard assembly procedure of FEM. Then global displacement solution is obtained. Implementation of multilevel X-DIC procedure is aptly explained in^24^ with several examples.

### Strain calculation using SPH procedure

Displacement vector **q**^*T*^ = {**u**, **r**} at each node is estimated by solving equation 5. Using Heaviside function *H*(*x*), displacement at node *i* is calculated as


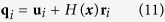


In order to estimate nodal strain, SPH approximation and interpolation function is used mainly for two reasons: (i) to smoothen displacement values at node *i* by interpolating with neighbouring nodal displacement values and (ii) to directly estimate nodal strain tensors using SPH functions. Here, the popular cubic spline function, proposed by Monaghan and Lattanzio, 1985^28^, is used as interpolation function which has the following form:


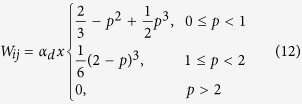


where *α*_*d*_ is the normalization factor, which is 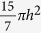 in two-dimensional space and *p* is the normalized distance between pixel *i* and *j*^*th*^ neighbor defined as 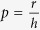 where *h* is the smoothing length. For this cubic spline function, the parameter *k*, which defines the effective area of support domain for node at **x** is defined by 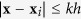, where *k* is a constant and in this analysis the value of *k* is taken as 2. For a 4 × 4 noded quadrilateral element, window size or support domain *kh* of 2 is assumed. The value of a function in a node is approximated with the values in nodes in its support domain as follows^29^:


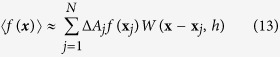


where Δ*A*_*j*_ is the area of influence of node *i* and 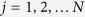 indicate nodes within the support domain of the node at **x**.

**Figure 1:**
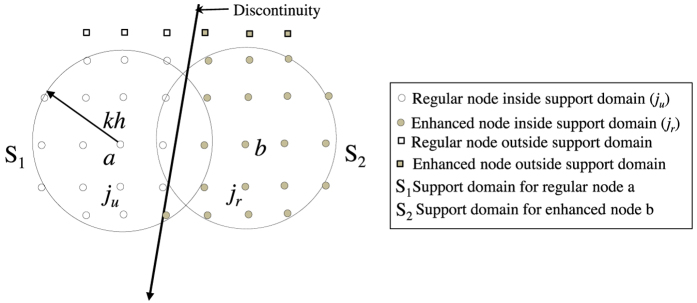
Schematic of the support domain of regular and enhanced node.

The strain tensors of a node are then obtained by using the following equation^29^:


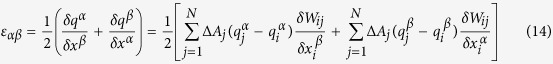


where *α* and *β* denote values of *x* and *y* components respectively. Here, *ε*_*xx*_, *ε*_*yy*_ and *ε*_*xy*_ are determined in Cartesian coordinates. It may be noted that, the support domain of a regular node may intersect a discontinuity as shown in Fig. 1. The nodes which only belongs to regular part (*j*_*u*_) will be the neighbour of a regular node *i* for estimating strain tensors. Similarly, an enhanced node can have neighbours which are both regular and enhanced. For that case, enhanced neighbours are only considered for estimating strains. It is clear that due to this restriction, equation 14 will have summation deficiency since 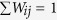. To overcome this deficiency, XSPH method is adopted as given in^30^.

### Experimental setup and description of sample materials

**Figure 2:**
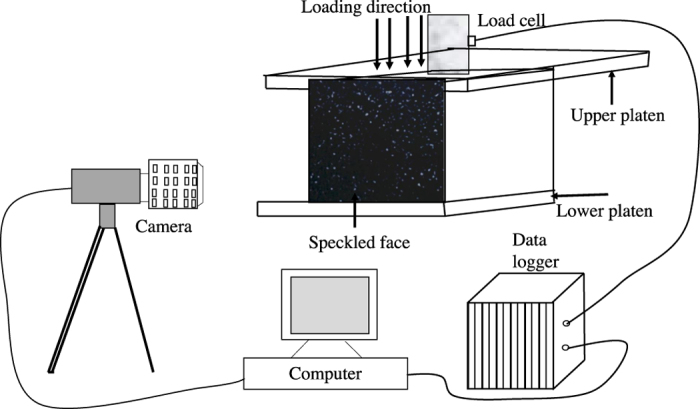
Schematic diagram of experimental setup.

Concrete as well as rock samples are tested in this study. Cubical samples of size of 150 mm are prepared and one vertical face of the sample is speckled using the procedure developed by Deb and Bhattacharjee^31^. A servo-controlled universal testing machine (UTM) of 3500 kN capacity is used to compress the experimental sample at a loading rate of 0.45 mm/min. A high precision data acquisition system mentioned in^32^ is used to capture the required data during experiment. The schematic diagram of the experimental set up is shown in Fig. 2. A Logitech carl zeiss tessar HD 1080P full HD web camera is used to seamlessly capture images of the speckled face with increment of loading.

Three concrete blocks of 150 mm sides and one sandstone rock sample of almost equal dimension are prepared and tested under uniaxial loading conditions. Two concrete samples (observation 1 and observation 2 mentioned below) are made as M40 concrete with characteristic compression strength of 40 MPa. The average elastic modulus of the samples is found to be 6.15 GPa. This is called “High Grade Concrete” and is used for construction of high rise buildings, vehicular pavements and others. The third concrete sample is generally termed as “High Performance Concrete” having grade M60 with characteristic compression strength of 60 MPa. The elastic modulus of the sample is found to be 7.3 GPa. This type of concrete is used in long bridge deck construction, piers, concrete dam, highway pavements for heavy vehicles and others. The design mix of the concrete is prepared in accordance to Indian Standard Code of practice, IS-456-2000. Sandstone rock sample is classified as sedimentary rock and generally found in the roof of a coal mine. The rock is used in this paper is of fine grained sandstone and is collected from a coal mine site. The compressive strength and elastic modulus of the sample are found to be 70.41 MPa and 7.3 GPa respectively.

## Results

### Experimental analysis: Observation 1

**Figure 3:**
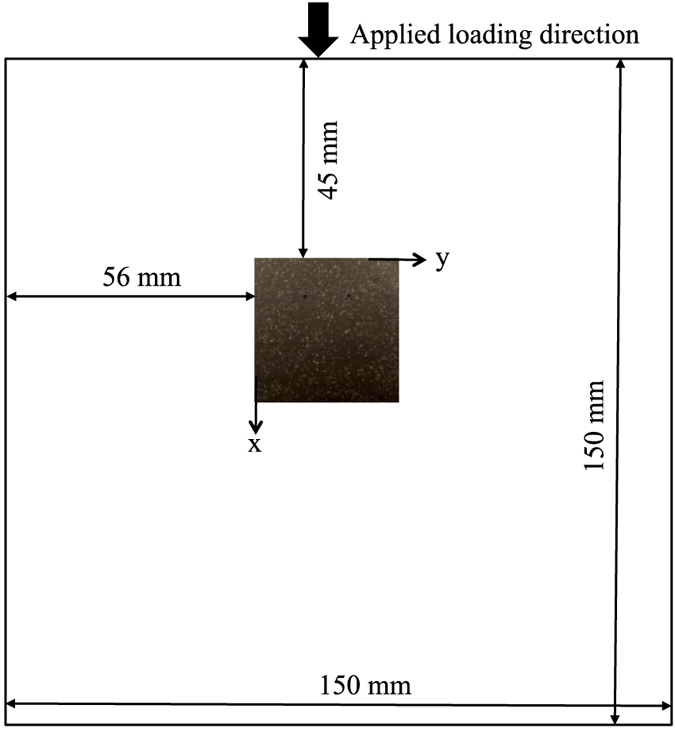
Position of image frame with respect to sample.

This section describes the development procedure of a pre-failure or yield indicator using results of an experiment conducted on a 150 mm cubical concrete sample. It may be noted that similar procedure is applied for the analysis of results of other samples and for validating the efficacy of the proposed indicator. In this experiment, 19 consecutive image frames having resolution of 600 × 600 are extracted at a time interval of 10 sec., meaning 0.075 mm of platen displacement at every 10 sec. The entire face of the sample shown in Fig. 3 has been speckled, however, the speckled portion, 600 × 600 pixels, shown in the figure is analyzed for determining yield indicator of the sample.

**Figure 4:**
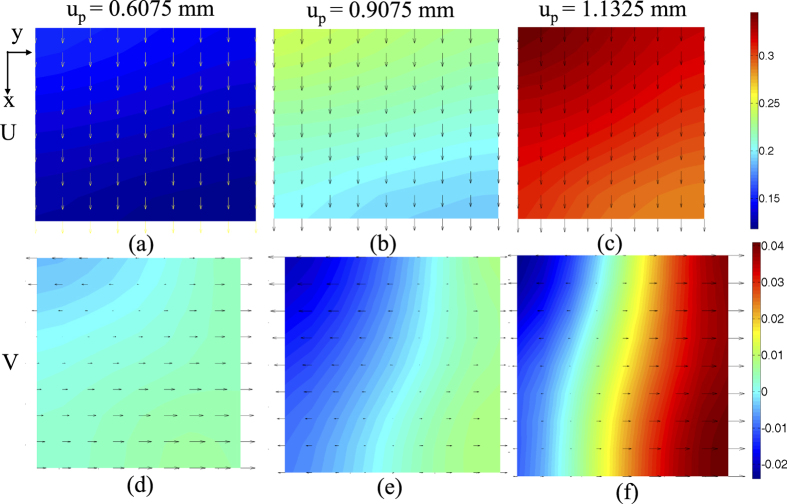
Displacement (*U*, *V* ) distributions with platen displacement.

Uni-axial compressive strength (UCS) of the sample is found to be 40.11 MPa at an axial strain of 9.05 × 10^−3^ (platen displacement of 1.3575 mm). X-DIC method is used to obtain the nodal displacements. Since there is no crack or discontinuity in the sample, displacement jump denoted by *r* in equation 11 is found to be negligible. Figure 4(a–f) show the displacement (*U*, *V*) distributions after elapsed time of 81 sec (platen displacement (*u*_*p*_) = 0.6075 mm, 45% of peak stress), 121 sec (*u*_*p*_ = 0.9075 mm, 77% of peak stress) and 151 sec (*u*_*p*_ = 1.1325 mm, 92% of peak stress). Figure 4(a–c) clearly show the increase in deformation along *x* -direction with the increment of platen displacement from the initiation of loading. Displacements along *y* - direction are found to be very less as compared to *U* displacements in *x* - direction (Fig. 4(d–f)).

**Figure 5:**
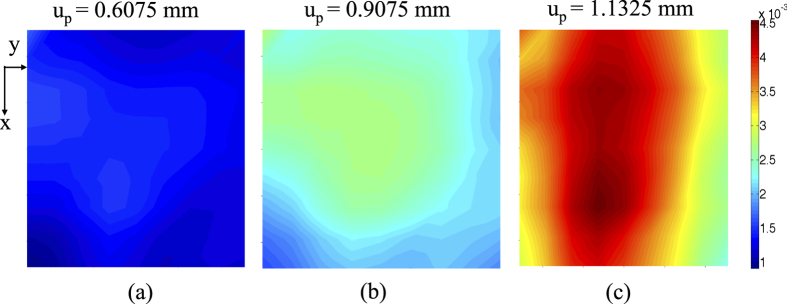
Cumulative effective strain distribution with platen displacement.

Strain tensors are estimated at each node using equation 14. In order to determine the variation in strains developed in the sample, effective strain (*ε*_*eff*_) is calculated using equation 15 for two consecutive image frames. Then cumulative effective strain is obtained upto *n*^*th*^ number of image pairs using equation 16. The cumulative effective strain is plotted at different time instants as shown in Fig. 5(a–c).


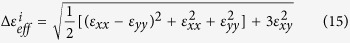



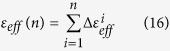


**Figure 6:**
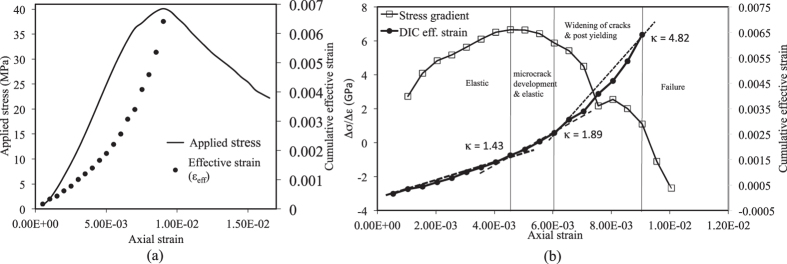
Observation 1: (**a**) Cumulative effective strain variations with axial strain; (**b**) Stress gradient variation with axial strain.

Figure 6(a) shows the cumulative effective strain and applied stress plot with axial strain. In this figure, effective strains of all nodes of the speckled area shown in Fig. 3 are averaged. The figure clearly shows that, effective stress as well as cumulative *ε*_*eff*_ change with axial strain. Mogi^26^, has showed using experimental results that slope of stress-strain curve decreases gradually with increase in strain and commented that in some cases, it becomes nearly constant at or beyond yield stress^26^. Figure 6(b) shows plots of stress gradient and cumulative effective strain of observation 1 sample with respect to axial strain. It can be seen that at the initial stage, stress gradient increases gradually and attains a peak value before it starts to drop. The fall in stress gradient may mark the onset of microcrack development in the sample. The average gradient of cumulative effective strain increases in microcrack development zone as compared to initial elastic zone. The rate of increase in cumulative effective strain further increases at around a point where stress gradient falls drastically marking the onset of yielding of the sample. As also observed by Mogi^26^ the sudden drop in stress gradient is the breaking or yield point in the stress-strain curve. It is also well known that at yielding, sample departs from elasticity and hence strain-path changes indicating pre-failure in the sample. The similar behaviour has been observed by Gramberg^33^ and he has commented that under uniaxial loading, microcracks initiate in rock samples at 50% of peak load and secondary fracture phenomena develops at 75% of peak load^33^.

**Figure 7:**
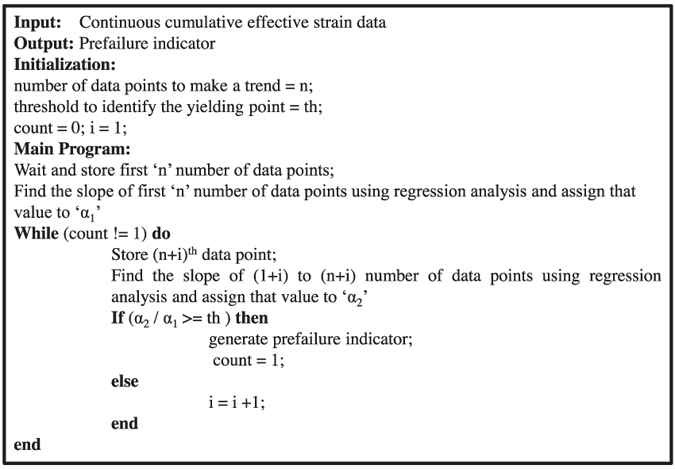
Algorithm for generating prefailure signature.

Beyond the yield point, the average gradient of cumulative effective strain is very high signifying post-yielding zone. In the same zone, stress gradient drops significantly until peak stress is attained or sample failure is occurred. In this paper, an algorithm is developed to identify onset of yielding as explained in Fig. 7.

In this algorithm an initial slope (*α*_1_) of strain is determined considering the first 5 data points. The 5 points are found to be suitable after analyzing several observations as explained later. Then next 5 data points are taken and slope (*α*_2_) of the line is determined and ratio of 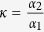, is obtained. The major idea is to find a value of *κ* such that stress is at around 75% of peak stress or a significant drop in stress gradient occurs. In this experiment, *κ* is found to be 1.89 when significant drop in stress gradient occurs (Fig. 6(b)). It will be shown later with other experimental results that in every case, *κ* ≥ 1.8 when the drop in stress gradient is significant and stress level in over 75% of peak stress. Therefore, in the algorithm, a threshold value of *κ* = 1.8 is assumed to mark the onset of yielding in the sample. Then the value of *κ* is checked with threshold (‘th’) for identifying yield indicator and the similar process continues. In the Fig. 6(b), *κ* values are also mentioned at various stages of sample behaviour.

**Figure 8:**
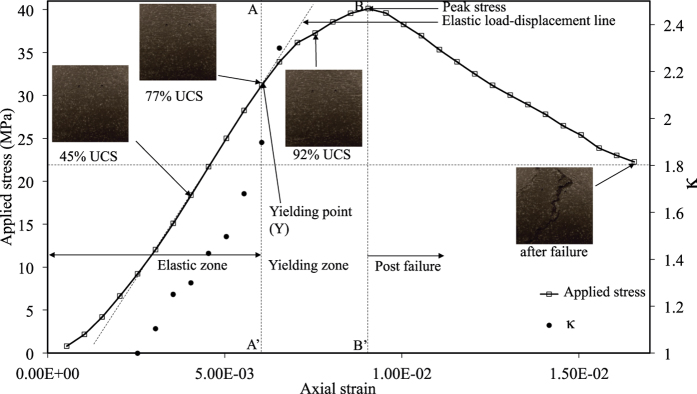
Observation 1: *κ* variation with axial strain.

Figure 8 shows axial stress and *κ* variation plots with axial strain. In this observation, the value of *κ* is found to be just above 1.8 (set as ‘th’ in Fig. 7) at an axial strain of 6.05 × 10^−3^ (platen displacement of 0.9075 mm) and applied stress of 31.19 MPa (77% of peak stress). At this point, a vertical line AA′ is plotted which meets horizontal line drawn at *κ* = 1.8. The zone of axial strain between 0 and AA′ is called ‘elastic zone’. It is noteworthy that a part of elastic zone also contains development of microcracks zone as shown in Fig. 6(b). The zone in between AA′ line and BB′ line (a vertical line at peak stress) is termed as ‘yield zone’. The zone beyond the peak stress is generally known as ‘post-peak’ or ‘failure zone’. The objective of this study is to find the ‘yield point’ in a non-contact manner, where permanent damage in the sample has occurred. The conditions of speckled surface at different loading conditions are also shown in the Fig. 8. It is difficult to notice any change in the sample by visual inspection of the images captured before failure. It is clear that the proposed algorithm has identified each zone adequately and identified the yield point which can be used as a prefailure indicator in material failure process. It can also be noticed that a major fracture develops at the middle of the sample. There is hardly any indication of this in the original images captured before failure. However, contours of the cumulative effective strain have clearly shown the indication of such a fracture as well as the position of it by the concentration of high strains (Fig. 5(b)).

In the next sections, another three experimental works are presented to show the repeatability of the proposed method for identifying yield point or prefailure indicator during sample failure process. Samples of observation 2 and 3 are made of concrete whereas sample 4 is a sandstone rock of approximately same dimension.

### Experimental analysis: Observation 2

**Figure 9:**
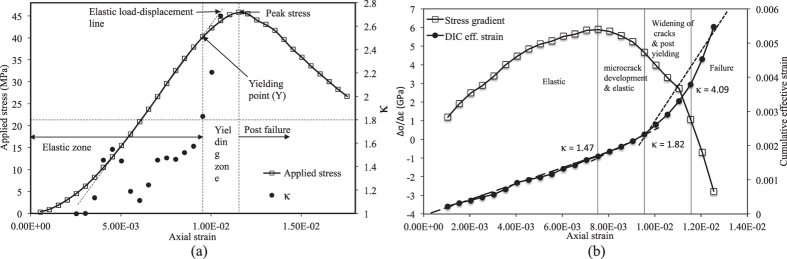
Observation 2: (**a**) *κ* variation with axial strain; (**b**) Stress gradient variation with axial strain.

In this experiment, 26 consecutive image frames having resolution of 1000 × 1000 are extracted at a time interval of 10 sec or 0.075 mm of platen displacement. The uni-axial compressive strength (UCS) of this sample is found to be 45.75 MPa at an axial strain of 1.155 × 10^−2^ (platen displacement of 1.7325 mm). Axial stress and *κ* are plotted with respect to axial strain as shown in Fig. 9(a). It can be noticed that once *κ* exceeds 1.8, yielding occurs in the sample. To confirm this finding, stress gradient and cumulative effective strain are plotted as before and shown in Fig. 9(b). As observation 1, it is clear that *κ* > 1.8 is definitely an indicator of yielding and *κ* ≈ 1.4 may signify onset of microcrack development in the sample. For this experiment, the yield point occurs at 40.21 MPa which is 87% of peak stress.

### Experimental analysis: Observation 3

**Figure 10:**
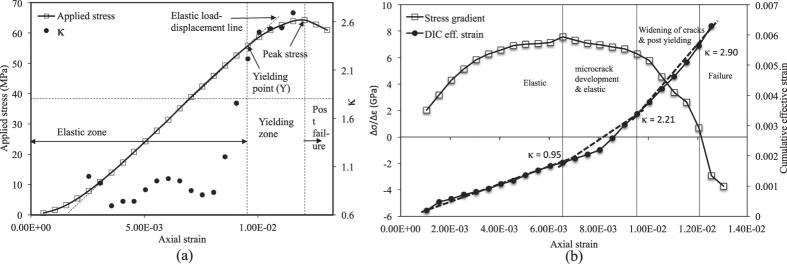
Observation 3: (**a**) *κ* variation with axial strain; (**b**) Stress gradient variation with axial strain.

The above findings are again validated with results of another experiment as described below. In this experiment, 25 consecutive image frames having resolution of 600 × 600 are extracted at a time interval of 10 sec. The uni-axial compressive strength (UCS) of this sample is found to be 64.21 MPa and it occurs at an axial strain of 1.205 × 10^−2^. Figure 10(a) plots *κ* and axial stress with respect to axial strain and shows that the value of *κ* remains near 1.0 until the stress approaches near to the yield value. Then the value of *κ* increases rapidly with axial strain and in the same token, stress gradient starts to fall significantly (Fig. 10(b)). This phenomena signify that microcrack development in the sample is not so severe at the early stage (after peak stress gradient) and as result, the sample carries almost 20 MPa more stress at failure. In this case, yielding occurs at *κ* = 2.21 whereas microcrack formation may have started at *κ* = 0.95. In this experiment, yield point occurs at an axial strain of 9.55 × 10^−3^ or at a stress level of 55.71 MPa (86% of peak stress).

### Experimental analysis: Observation 4

**Figure 11:**
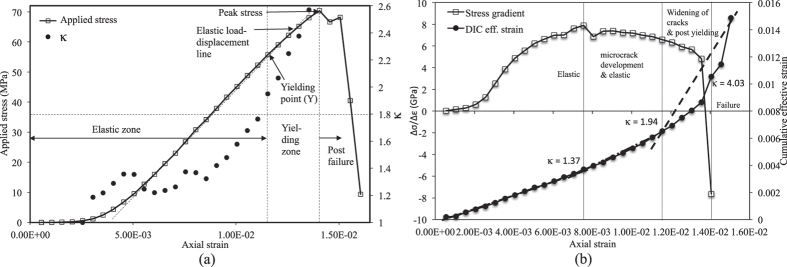
Observation 4: (**a**) *κ* variation with axial strain; (**b**) Stress gradient variation with axial strain.

Similar experiment is repeated for a standstone rock sample having length 150 mm, width 165 mm and thickness 145 mm. In this case, 29 consecutive image frames having resolution of 600 × 400 are extracted at a time interval of 10 sec. The uni-axial compressive strength (UCS) of rock sample is found to be 70.41 MPa as shown in Fig. 11(a). The concept developed in this paper is validated with results obtained from this experiment. Figure 11(b) clearly manifests the onset of microcrack development as well as yielding of the sample. The results confirm that yield point occurs at *κ* = 1.94 and microcrack development in the sample may begin at *κ* = 1.37. Sandstone sample yields at axial strain of 1.155 × 10^−2^ or 79% of peak stress (55.75 MPa).

**Figure 12:**
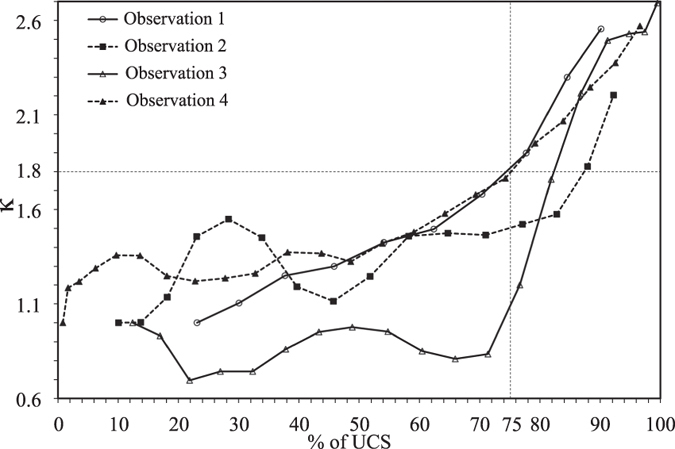
Summary of *κ* variation with % of UCS.

The summary of the above results is presented in Fig. 12 and listed in Table 1. Figure 12 shows the plot between *κ* and axial stress in terms of percentage of peak stress for all 4 observations. It can be seen that the trend of the curves are almost similar especially after the stress on the samples exceeds 75% of peak stress. It is certain that response of the samples to uniaxial loading after 75% of peak stress or *κ* > 1.8 changes drastically signifying softening behaviour. This is a clear indication that the samples have departed from elasticity.

### Discussion on *κ*

With the help of four examples, it is shown that gradient of axial stress i.e. 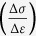 starts to decrease at about 55 to 65% of peak stress for concrete blocks and 38% for the rock block. Several authors have also noticed this phenomenon and suggested that this may happen due to the development of microcracks in the sample^26^,^33^. The study also points to the fact that at around 77 to 86% of the peak stress for concrete blocks and at 79% of peak stress for the rock block, stress gradient 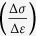 falls rapidly meaning yielding in the sample or coalescence of microcracks to form shear band(s) in the sample. Recently, a numerical study based on Smoothed Particle Hydrodynamics (SPH) is conducted in a rock sample under uniaxial loading condition^34^. Authors have shown that microcracks are developed in the sample at around 50% of peak stress and they coalescence to form shear bands at around yield stress^34^.

In this study, an attempt is made to identify two important events in rock failure mechanism, viz. development of microcracks and yielding, using FEM-DIC procedures and with the developed indicator *κ*. In the first case, *κ* is found to be around 1.4 (except for one sample) and for yielding, it is generally over 1.8. Since the indicator is developed based on strain, it signifies the increment of sample volume with loading. The volumetric strain is a measure of change of volume with respect to the original volume and for uniaxial loading condition, it can be given as


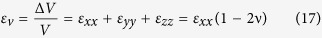


where *ε*_*xx*_, *ε*_*yy*_ and *ε*_zz_ are normal strain in *x*, *y* and *z* axis directions respectively, loading direction in *x*-direction and ν denotes Poisson’s ratio of the material. It may also be noted that volumetric strain is also a measure of amount of microcracks developed in the sample during loading process and hence signifies to the dilatancy of the sample. In this respect, the indicator *κ* can be considered as a measure of volume increment in the sample with respect to its original volume and hence (*κ* − 1) is proportional to volumetric strain as given below:


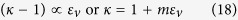


where *m* is the proportionality constant.

**Figure 13:**
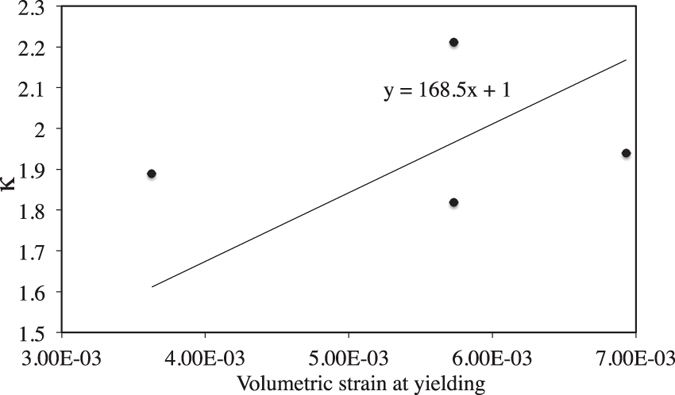
Relationship between *κ* and volumetric strain of the experimental samples.

Experimental results suggest that yielding may occur at axial strain as low as 0.006 and as high as 0.011. Considering lower and upper limits of axial strain as 0.005 and 0.015 respectively at yielding and Poisson’s ratio of concrete/rock material varies within 0.1 to 0.3, lower and upper bound of proportionality constant are found to be in the range of 66.67 and 400. For the given samples, proportionality constant is found to be 168.5 for ν = 0.2 (Fig. 13).

## Conclusion

Development of a prefailure indicator has great importance for providing early warning of stability of structures made in rock mass. In this research work, X-DIC method is applied for measuring displacements in a non-contact manner those may be developed in geomaterials during loading process. The concept of SPH is introduced to compute the nodal strain tensors. Cumulative effective strain data are used to develop a pre-failure indicator which is capable of predicting stress level for microcrack development as well as yielding in samples during loading process. A new algorithm is developed to automatically identify yield point as the loading progresses on the sample. This paper describes the applicability of the proposed algorithm by conducting four experiments. It is found that *κ* > 1.8 is a clear indication of yielding meaning that material response to loading has departed from elasticity. In the same token, *κ* ≈ 1.4 signifies development of microcracks in the sample. Using these concepts, yielding of any structure may be ascertained in a non-contact manner and it shows a tremendous potential for field applications. Moreover, the study finds that the parameter *κ* may be related to the change of volume of the sample during loading and points to volumetric strain. This threshold values also turn out to be the indicators of 75% and 50% of peak stress which are normally considered as yield and microcrack development points respectively.

## Additional Information

**How to cite this article**: Bhattacharjee, S. and Deb, D. Optical strain based pre-failure indication in failure process of geomaterials. *Sci. Rep.* **6**, 24741; doi: 10.1038/srep24741 (2016).

## Figures and Tables

**Table 1:** Summary of strain based pre-failure signature.

Observation no.	UCS (MPa)	*κ* value at yielding point	Axial strain at yielding (×10^−3^)	% UCS at yielding point
1	40.11	1.89	6.05	77
2	45.75	1.82	9.55	87
3	64.21	2.21	9.55	86
4	70.41	1.94	11.55	79
